# Digital Health Literacy and Attitudes Toward Telehealth Use in Practice Among Nursing Students in Saudi Arabia: Cross-Sectional Study

**DOI:** 10.2196/94722

**Published:** 2026-06-23

**Authors:** Muna Alharbi, Ahmed S Alharthi, Abdulrahman A Azab, Renad Alharthi

**Affiliations:** 1 Community Nursing and Healthcare Department, Faculty of Nursing, Umm Al-Qura University, Al-Abdiyyah Campus, Taif Road, Makkah, 24381, Saudi Arabia; 2 East Hawiyah Primary Health Care Centre, Taif Health Cluster, Taif, Saudi Arabia; 3 Hera General Hospital, Makkah Health Cluster, Makkah, Saudi Arabia; 4 Maternity and Children Hospital, Makkah Health Cluster, Makkah, Saudi Arabia

**Keywords:** attitude, digital literacy, nursing education, nursing students, practice, telehealth, telemedicine

## Abstract

**Background:**

Nursing students are the future workforce, and their readiness to use digital health tools is important. Previous studies have focused on knowledge and attitudes; however, they have not examined the wide range of digital health literacy levels that may influence nursing students’ attitudes toward using telehealth in clinical settings.

**Objective:**

This study aimed to determine the relationship between nursing students’ digital health literacy and their attitudes toward telehealth use in practice.

**Methods:**

A cross-sectional design was used. The sample consisted of undergraduate nursing students enrolled in a Bachelor of Nursing program at a selected Saudi Arabian university. The online survey used 2 scales: the Digital Health Care Literacy Scale and the Nurses’ Attitudes Toward Use of a Telehealth Scale.

**Results:**

A total of 273 students participated (mean age 21.3, SD 1.9 years). Most of the nursing students demonstrated a high digital health literacy level (n=184, 67.4%; mean Digital Health Care Literacy Scale score 11.9 out of 15, SD 3.1). Digital health literacy was a significant predictor of positive attitudes toward telehealth use in practice (adjusted odds ratio 1.48, 95% CI 1.28-1.71; *P*<.001). Male students were significantly less likely to report positive attitudes than female students (adjusted odds ratio 0.62, 95% CI 0.39-0.97; *P*=.03). However, academic year, telehealth workshops, and informatics courses were not significantly associated with positive attitudes toward telehealth use in practice.

**Conclusions:**

Higher levels of literacy appear to correlate with more positive attitudes toward telehealth use in practice. However, current formal education and workshops had no apparent influence on digital health literacy. This suggests a potential need for strengthening digital training and development in nursing education. This may enhance telehealth readiness and support future digital health care delivery.

## Introduction

The World Health Organization defines telehealth as the provision of health care services to individuals at a distance through information and communication technologies. It enables care to be delivered when the provider and the patient are in different physical locations [1]. Telehealth uses communication technologies for diagnosing and treating diseases, supporting research and assessment, and providing continuous professional education [1]. The rapid expansion of telehealth has transformed the health care system nationally and internationally. This expansion has increased the demand for professional competencies in digital health technologies.

Digital health literacy refers to the ability to access, analyze, and apply health information via electronic means to address health-related issues [2,3]. It also includes the ability to apply telehealth services and maintain electronic communication with health care professionals [2,3]. As telehealth becomes increasingly integrated into health care systems, digital health literacy has become an important competency for nursing students and future health care professionals. Students with stronger digital competencies may engage more effectively with telehealth technologies and adapt more easily to digital health care environments [2,4].

Previous studies have reported moderate to high levels of digital health literacy among nursing students internationally. A study among Indonesian nursing students found that participants possessed fundamental digital health skills. These skills were influenced by factors including higher self-perceived internet skills and more frequent use of the internet for health-related purposes [5]. Similarly, a study from Turkey reported good digital health literacy among nursing students, with higher scores observed among older students and those who reported greater internet use [6]. In contrast, a study of nursing students in Ethiopia found that students had lower digital health literacy levels, particularly among first-year students and those from rural areas [7]. These findings suggest that digital health literacy may vary according to educational exposure, demographic factors, and access to digital technologies [5-7].

Studies conducted in Saudi Arabia have also revealed positive attitudes and moderate knowledge and awareness of telehealth among nursing students [8,9]. This highlights the need for educational programs that strengthen students’ digital health competencies [9]. Similar findings were reported in Egypt, where nursing students show positive attitudes toward telehealth despite limited formal training [10,11]. In South Korea, nursing students also had limited direct exposure to telehealth education and clinical telehealth experiences [12]. Nevertheless, telehealth education and self-efficacy were associated with more positive attitudes toward telehealth use in practice [12].

Telehealth has undergone rapid expansion and transformation in Saudi Arabia. In alignment with Vision 2030, the Ministry of Health has launched the E-Health Initiative in Digital Transformation to improve the health care system’s effectiveness and efficiency through information technology and digital transformation [13]. According to the Technology Acceptance Model, individual adoption of new technologies is influenced by perceived usefulness and ease of use [14,15]. In the context of telehealth, digital health literacy may contribute to these perceptions by improving individuals’ confidence and ability to use digital technologies effectively [2-4].

Although previous studies have investigated nursing students’ knowledge and attitudes regarding telehealth, digital health literacy and digital health knowledge are conceptually different and often used interchangeably. Digital health knowledge refers to an individual’s understanding of what digital health is; when it is used; and the benefits, limitations, and associated ethical or legal considerations [1,16,17]. Digital health literacy, however, goes beyond awareness to encompass the practical ability to access, use, and assess telehealth services effectively in actual practice [2-4]. Whereas knowledge appears primarily cognitive and content-based, literacy is skill-based and reflects the capacity to apply that knowledge in real-world telehealth interactions [2,3]. Thus, a person may possess knowledge about telehealth without necessarily having the literacy required to use it competently.

Despite increasing integration of telehealth into health care education and practice, there is a lack of evidence regarding the relationship between nursing students’ digital health literacy and their attitudes toward telehealth use in practice. Therefore, this study aimed to examine the relationship between digital health literacy and attitudes toward telehealth use in practice among undergraduate nursing students in Saudi Arabia. The study also explored demographic and educational factors associated with positive attitudes toward telehealth use in practice.

## Methods

### Design

A cross-sectional study was conducted to capture undergraduate nursing students’ digital health literacy and attitudes toward telehealth use in practice at a single point in time. This study was reported according to the STROBE (Strengthening the Reporting of Observational Studies in Epidemiology) statement guidelines (Checklist 1).

### Participants and Setting

This study focused on students enrolled in a Bachelor of Nursing program within a selected university in western Saudi Arabia. In this selected program, telehealth was introduced theoretically to nursing students in many courses; however, limited practical opportunities were available in clinical settings. A convenience sampling method was applied to recruit the participants, which included distribution of invites through social media platforms, emails, and announcements sent through the university’s learning management system. To prevent duplicate responses, only 1 submission per device was allowed.

The inclusion criteria for the study were current nursing students in their second year to internship year. First-year students were excluded because they had not yet progressed into the specialized nursing curriculum and had limited exposure to clinical courses, nursing informatics, or telehealth-related learning experiences. Therefore, only second-year, third-year, fourth-year, and internship students were included to ensure that participants had sufficient academic and clinical exposure to meaningfully evaluate digital health literacy and attitudes toward telehealth use in practice. Licensed or registered nurses enrolled in bridging or postlicensure programs were excluded because prior professional experience may influence digital health literacy and attitudes toward telehealth use in practice independently of undergraduate nursing education.

The data were collected from November 2025 to January 2026. During this research period, 440 female and 223 male nursing students were enrolled in the Bachelor of Nursing program. The targeted sample size was 244, which met the required sample size, with a 95% CI and 5% margin of error.

### Data Collection Instrument

The survey consisted of 3 sections: part 1 asked about demographic data, while the second and third parts used 2 scales adopted from previously published and publicly accessible studies [18,19]. The first part of the survey collected demographic data such as sex, age, and academic year (second, third, fourth, or internship). Four questions inquired whether students had attended workshops or lectures related to telehealth, had taken nursing informatics courses, understood the definition of telehealth, and had applied telehealth in clinical placements.

The second part of the survey assessed digital health literacy using the Digital Health Care Literacy Scale (DHLS), with 3 items adopted from a previously published and publicly accessible study [18]. The scale exploratory factor analysis demonstrated good psychometric properties, with Cronbach α of 0.90 and 78% explained variance, supporting the scale’s validity and reliability [18]. The responses were measured using a 5-point Likert scale ranging from strongly disagree to strongly agree.

The third part of the survey assessed the students’ attitudes toward telehealth in practice using the 19-item Nurses’ Attitudes Toward Use of a Telehealth Scale (NATUTS), which was obtained from a previously published and publicly accessible study [19]. NATUTS measured 3 factors: satisfaction, rejection, and development. The instrument demonstrated strong psychometric properties, with Cronbach α values ranging from 0.86 to 0.93 and a total explained variance of 64.4%, supporting its validity and reliability for assessing attitudes toward telehealth [19]. For descriptive interpretations purposes, the items were conceptually grouped under 4 domains, namely perceived benefits, professional integration and reliability, negative emotions and resistance, and engagement and self-development. Responses were recorded using a 5-point Likert scale ranging from strongly disagree to strongly agree.

The survey was administered in English because English is the language of nursing instruction at the study institution, and translation was therefore not needed. The survey was checked for face and content validity to assess its clarity and relevancy to the study objective before distribution. It was sent to 3 experts in nursing education and digital health for feedback. A pilot test was conducted with 10 nursing students to evaluate clarity, readability, and completion time. No modifications were required based on their feedback. Pilot participants were excluded from the final analysis.

The 3 DHLS items were adopted to assess the level of digital health literacy of the students. Each item was rated using the 5-point Likert scale, ranging from 1 (strongly disagree) to 5 (strongly agree). The 3 items were rated accordingly, and the total DHLS scores ranged from 3 to 15. The digital health literacy score was classified accordingly: 3 to 7 (low), 8 to 11 (moderate), and 12 to 15 (high). The total NATUTS score was calculated by adding up all 19 items after negatively worded items were reverse-coded to investigate predictors of positive attitudes toward telehealth. The total NATUTS scores ranged from 19 to 95. Higher scores indicated more positive attitudes toward telehealth use in practice. The sample mean score was used as a cutoff to dichotomize the overall attitude score. Mean score dichotomization has been used to facilitate interpretation of logistic regression outcomes when no established cutoff exists. Values below the mean were classified as negative attitudes, and scores equal to or above the mean were classified as positive attitudes. The multivariable logistic regression model was then fitted with the dichotomized variable.

### Data Analysis

SPSS (version 28; IBM Corp) was used to perform all statistical analyses. Descriptive statistics were adopted to classify the nursing students’ educational background, level of digital health literacy, sociodemographic attributes, and attitude toward telehealth use in practice. Frequencies and percentages denote the categorical variables, and means and SDs denote the continuous variables.

To examine factors associated with positive attitudes toward telehealth use in practice, multivariable logistic regression analysis was performed. Digital health literacy score, age, sex, academic year, exposure to telehealth education, completion of a nursing informatics course, and telehealth use during clinical placement were entered as independent variables in the model. Adjusted odds ratios (AORs) with 95% CIs were calculated. A *P* value of <.05 was considered statistically significant.

### Ethical Considerations

The study adhered to ethical research standards and received approval by a relevant institutional review board of Umm Al-Qura University (receipt number HAPO-02-K-021-2025-5-2785). A statement at the beginning of the survey informed participants of the study’s objectives, processes, possible benefits, and any related risks. It explained that participants’ identities would remain anonymized and protected.

Participants provided consent by completing and submitting the survey anonymously. There were no significant risks, and the only cost to the participants was the time taken to complete the survey. Participation was voluntary, and responses were collected anonymously. No financial compensation or incentives were provided to participants for taking part in the study.

## Results

Table 1 presents the sociodemographic and academic characteristics of the participating nursing students (N=273). Most students were aged 21 and 22 years (160/273, 58.6%), followed by 19 and 20 years (82/273, 30%), with a mean age of 21.3 (SD 1.9) years. Most participants were female (209/273, 76.6%). Regarding academic level, most students were in their third year (101/273, 37%), followed by fourth-year students (95/273, 34.8%), nursing interns (45/273, 16.5%), and second-year students (32/273, 11.7%). A high proportion of students reported having attended a telehealth workshop or lecture (198/273, 72.5%) and completing a nursing informatics course (194/273, 71.1%). The vast majority reported knowing the meaning of telehealth (244/273, 89.4%), and more than half reported using telehealth during their clinical placement (148/273, 54.2%).

**Table 1.** Sociodemographic and academic characteristics of nursing students (N=273).

Variables	Nursing students
Age (years), n (%)	
19‐20	82 (30)
21‐22	160 (58.6)
>23	31 (11.4)
Age (years), mean (SD)	21.3 (1.9)
Sex, n (%)	
Male	64 (23.4)
Female	209 (76.6)
Academic year, n (%)	
Second year	32 (11.7)
Third year	101 (37)
Fourth year	95 (34.8)
Nursing intern	45 (16.5)
Have you attended a workshop or lecture about telehealth?, n (%)	
Yes	198 (72.5)
No	75 (27.5)
Have you taken a nursing informatics course?, n (%)	
Yes	194 (71.1)
No	79 (28.9)
Do you know the meaning of telehealth?, n (%)	
Yes	244 (89.4)
No	29 (10.6)
During your clinical placement, do you use telehealth?, n (%)	
Yes	148 (54.2)
No	125 (45.8)

Table 2 illustrates the distribution of nursing students’ responses to the 3-item DHLS. More than half (140/273, 51.3%) strongly agreed that they could use applications or programs such as Zoom independently, while 77 (N=273, 28.2%) agreed. Similarly, 130 (N=273, 47.6%) students strongly agreed and 75 (27.5%) agreed that they could set up video chats without assistance. Regarding problem-solving skills, 91 (N=273, 33.3%) students agreed and 80 (29.3%) strongly agreed that they could resolve basic technical issues on their own, while a smaller proportion (65/273, 23.8%) remained neutral. The overall DHLS score ranged from 3 to 15, with a mean of 11.9 (SD 3.1) out of 15.

**Table 2.** Distribution of nursing students’ responses to the Digital Health Care Literacy Scale items (N=273).

Digital Health Care Literacy Scale items^a^	Strongly disagree, n (%)	Disagree, n (%)	Neutral, n (%)	Agree, n (%)	Strongly agree, n (%)
I can use applications or programs (such as Zoom) on my cell phone, computer, or another electronic device on my own (without asking for help from someone else).	16 (5.9)	13 (4.8)	27 (9.9)	77 (28.2)	140 (51.3)
I can set up a video chat using my cell phone, computer, or another electronic device on my own (without asking for help from someone else).	15 (5.5)	20 (7.3)	33 (12.1)	75 (27.5)	130 (47.6)
I can solve or figure out how to solve basic technical issues on my own (without asking for help from someone else).	12 (4.4)	25 (9.2)	65 (23.8)	91 (33.3)	80 (29.3)

a Range 3-15; mean 11.9, SD 3.1.

The overall distribution of nursing students’ digital health literacy levels. Most participants had a high level of digital health literacy (184/273, 67.4%). Exactly 61 (22.3%) students had a moderate level, and a smaller proportion of students (28/273, 10.3%) showed a poor level of digital health literacy.

Table 3 presents the distribution of nursing students’ attitudes toward telehealth use in practice. Overall, the findings show a predominantly positive attitude. In the perceived benefits of telehealth domain, most agreed or strongly agreed that telehealth reduces nurses’ workload (211/273, 77.3%), is an important part of nursing services (218/273, 79.9%), is essential in nursing (204/273, 74.7%), improves individuals’ quality of life (232/273, 85%), simplifies patient-nurse communication (210/273, 76.9%), enables nursing services to be carried out more effectively (209/273, 76.6%), gives professional advantage (202/273, 74%), and eases nurses’ workloads (221/273, 80.9%). In the professional integration and reliability domain, 216 (N=273, 79.1%) students agreed or strongly agreed that they would like to include telehealth in professional practice, 173 (63.3%) considered it reliable, and 209 (76.5%) viewed it as essential in nursing services. In the negative emotions and resistance domain, agreement was much lower, indicating limited reluctance: only 61 (22.4%) reported disliking telehealth, 69 (25.3%) felt nervous using it, 67 (24.5%) experienced conflict with its use, 100 (36.7%) used it because they had to, and 71 (26%) would not recommend it to colleagues. Regarding the engagement and self-development domain, over half agreed or strongly agreed that they follow current telehealth developments (135/273, 49.5%), improve themselves regarding telehealth (167/273, 61.2%), and research telehealth (129/273, 47.3%). The overall NATUTS score ranged from 19 to 95, with a mean of 70.93 (SD 11.52) out of 95.

**Table 3.** Distribution of nursing students’ attitudes toward telehealth use in practice (N=273).

Statements^a^	Strongly disagree and disagree, n (%)	Neutral, n (%)	Strongly agree and agree, n (%)
1. Perceived benefits of telehealth			
I believe that telehealth reduces nurses’ workloads.	28 (10.3)	34 (12.5)	211 (77.3)
I believe telehealth is an important part of nursing services.	21 (7.7)	34 (12.5)	218 (79.9)
Telehealth is an essential part of nursing.	27 (9.9)	42 (15.4)	204 (74.7)
I believe that telehealth is effective in improving individuals’ quality of life.	19 (7)	22 (8.1)	232 (85)
Telehealth simplifies patient-nurse communication.	22 (8)	41 (15)	210 (76.9)
Telehealth enables nursing services to be carried out more effectively.	23 (8.4)	41 (15)	209 (76.6)
Telehealth gives me an advantage in my profession.	24 (8.8)	47 (17.2)	202 (74)
I believe telehealth eases nurses’ workloads.	23 (8.4)	29 (10.6)	221 (81)
2. Professional integration and reliability			
I would like to include telehealth in professional practices.	26 (9.5)	31 (11.4)	216 (79.1)
Telehealth is reliable.	24 (8.8)	76 (27.8)	173 (63.4)
Telehealth is essential in nursing services.	27 (9.9)	37 (13.6)	209 (76.6)
3. Negative emotions and resistance			
I don’t like using telehealth.	157 (57.5)	55 (20.1)	61 (22.3)
I get nervous when using telehealth.	138 (50.5)	66 (24.2)	69 (25.3)
I have a conflict with using telehealth.	146 (53.4)	60 (22)	67 (24.5)
I use telehealth because I have to.	78 (28.6)	95 (34.8)	100 (36.6)
I would not recommend telehealth to my colleagues.	156 (57.1)	46 (16.8)	71 (26)
4. Engagement and self-development			
I follow current developments regarding telehealth.	49 (17.9)	89 (32.6)	135 (49.5)
I research telehealth.	57 (20.9)	87 (31.9)	129 (47.3)
I improve myself regarding telehealth.	32 (11.7)	74 (27.1)	167 (61.2)

a Mean 70.93, SD 11.52; range 19-95.

Multivariable logistic regression analysis was conducted to determine whether digital health literacy predicted positive attitudes toward telehealth use in practice after controlling for demographic and educational variables. Digital health literacy was a significant predictor of positive attitudes toward telehealth use in practice (AOR 1.48, 95% CI 1.28-1.71; *P*<.001). Male students were significantly less likely to report positive attitudes compared with female students (AOR 0.62, 95% CI 0.39-0.97; *P*=.03). No significant associations were observed for age, academic year, telehealth workshops, informatics courses, or telehealth use during clinical placement (Table 4).

**Table 4.** Multivariable logistic regression predicting positive attitudes toward telehealth use in practice (N=273).

Variables	AOR^a^ (95% CI)	*P* value^b^
Digital health literacy score	1.48 (1.28‐1.71)	<.001
Male (vs female)	0.62 (0.39‐0.97)	.03
Age (years)		
21 and 22 (vs 19 and 20)	0.91 (0.63‐1.32)	.62
≥23 (vs 19 and 20)	0.74 (0.40‐1.36)	.33
Academic year		
Third year (vs second year)	1.18 (0.64‐2.19)	.60
Fourth year (vs second year)	1.32 (0.70‐2.50)	.39
Intern (vs second year)	1.01 (0.52‐1.95)	.98
Telehealth workshop (yes vs no)	1.05 (0.66‐1.67)	.84
Informatics course (yes vs no)	1.12 (0.71‐1.78)	.62
Telehealth use in clinical placement (yes vs no)	1.09 (0.70‐1.69)	.69

a AOR: adjusted odds ratio.

b Significance level: *P*<.05.

## Discussion

### Principal Findings

The purpose of this study was to examine the relationship between digital health literacy and attitudes toward telehealth use in practice among nursing students. The findings demonstrated that high digital health literacy was significantly associated with positive attitudes toward telehealth use in practice. Students with higher digital health literacy were significantly more likely to perceive telehealth as beneficial, reliable, and relevant for nursing practice.

Most of the nursing students demonstrated a high level of digital health literacy. This result aligns with previous studies that found moderate to high digital health literacy among nursing and health sciences students [20,21]. However, the findings should be interpreted with caution. Although students may possess the ability to access and use digital health information, this does not necessarily indicate preparedness to apply telehealth in complex clinical situations. Previous studies have shown that nursing students often experience difficulties in critically evaluating online health information and in applying digital knowledge in clinical decision-making [20,21]. Thus, possessing digital health literacy skills alone may be insufficient without practical telehealth training and supervised clinical application.

Most of the students stated that telehealth decreases workload, improves the quality of life of patients, and plays a critical role in nursing practice. These results are consistent with those of previous studies demonstrating that nursing students perceive telehealth as beneficial to health care accessibility, efficacy, and communication [19,22]. Positive attitudes toward telehealth use in practice may reflect nursing students’ increasing exposure to digital technologies in both educational and health care settings. These attitudes may also be influenced by the rapid expansion of telehealth services following the COVID-19 pandemic [6,20]. Thus, students may perceive telehealth as consistent with contemporary nursing roles which require digital communication and remote patient monitoring [4,22].

Despite the reported positive attitudes, some students exhibited negative feelings such as reluctance and nervousness. These findings are important because they show that positive attitudes can sometimes coexist with discomfort, limited confidence, or perceived external pressure to use digital technologies. This may indicate that some students accept telehealth while still lacking confidence in their practical ability to use it efficiently and effectively. Such findings align with those of previous research, where the nursing students were hesitant due to reliability concerns and insufficient practical exposure [12,23]. These findings suggest that telehealth education should include hands-on simulation and supervised practice experiences to improve confidence and reduce resistance.

The presence of negative emotions toward telehealth use in practice may reflect limitations of current nursing education strategies. Although many students attended telehealth workshops or completed informatics-related courses, these experiences did not lead to more positive attitudes toward telehealth use in practice. This finding suggests that current educational strategies may emphasize theoretical knowledge more than practical competency. Short workshops or isolated informatics courses may increase awareness of telehealth without sufficiently improving students’ confidence, communication skills, or ability to integrate telehealth into patient care [23]. Subsequently, students may recognize the importance of telehealth while experiencing anxiety or resistance to its practical implementation.

Digital health literacy independently predicted students’ attitudes toward telehealth use in practice even after controlling for demographic and educational variables. This finding suggests that digital health literacy is not only a technical skill but also a determinant of technology acceptance in health care settings. Students who are confident in using digital tools may perceive telehealth systems as easier to use and thus more useful in clinical care. These results align with the Technology Acceptance Model, which suggests that individuals with higher perceived competence in technology are more likely to adopt digital innovations in practice [14,15]. However, students with lower literacy levels may experience uncertainty and resistance due to difficulties navigating digital health care environments. These findings suggest that digital competencies play a critical role in shaping students’ readiness to adopt telehealth technologies in future clinical practice. As telehealth becomes increasingly integrated into health care systems globally, digital health literacy may represent an essential professional competency for future nurses [4]. Therefore, improving digital health literacy may help not only to develop technical competence but also to reduce anxiety and strengthen professional acceptance of telehealth technologies.

The study statistically found that male students were less likely to report positive attitudes toward telehealth use in practice than female students. Although evidence regarding sex differences in attitudes toward telehealth among nursing students remains limited, this result may be related to communication preferences, engagement with patient-centered care, or attitudes toward health care technologies. However, this result should be interpreted cautiously given the lack of literature available and the potential influence of cultural and educational factors. Additional research is needed to better understand the reasons underlying these differences.

Overall, the results imply that telehealth education in nursing programs needs to be further enhanced beyond theoretical instruction. While students generally demonstrated positive attitudes and high digital health literacy, the persistence of nervousness, reluctance, and feelings of obligation indicates gaps between knowledge and practical readiness. Nursing curricula may benefit from integrating experiential telehealth learning activities such as simulations, virtual consultations, case-based learning, and supervised clinical telehealth experiences. These approaches may help students develop greater confidence, reduce resistance, and improve their ability to apply telehealth competently in real health care settings.

To summarize, digital health knowledge reflects understanding, whereas digital health literacy reflects the capacity to apply that understanding in practice. This study contributes to existing evidence that digital health literacy plays an important role in technical competence and is positively associated with students’ attitudes toward telehealth use in practice. Nursing education courses need to strengthen digital competencies to reduce resistance and promote more positive perceptions of telehealth within nursing. As telehealth becomes integrated into health care systems worldwide, preparing digitally competent nursing graduates is critical to future workforce readiness.

### Limitations

The cross-sectional design and single-educational setting of this study make it difficult to draw conclusions about the causal relationships between students’ attitudes toward telehealth use in practice and their level of digital health literacy. It was not possible to ascertain whether exposure to telehealth education results in long-lasting changes in attitudes or abilities because the data were gathered all at once. Additionally, the results are less applicable to other nursing programs with distinct curricula, resources, or student characteristics when the study is limited to a single institution. Consequently, findings should be interpreted cautiously, as they might not be generalizable to the broader population of undergraduate nursing students because of potential self-reported bias.

### Conclusions

The findings of this study demonstrate that nursing students have high levels of digital health literacy. In addition, most of the students exhibit positive attitudes toward telehealth use in practice, particularly pertaining to patient care delivery, decreasing workload, and professional integration. The significant finding was the association between higher digital health literacy levels and positive attitudes toward telehealth use in practice. These findings suggest a potential need to strengthen digital health content within nursing curricula to strengthen nurses’ digital skills through formal training and continuous professional development.

Educators and clinical leaders can modify training strategies to meet the needs of specific groups. This targeted approach may reduce disparities in digital health and telehealth competencies across the clinical workforce. Offering workshops or informal learning experiences does not guarantee improved competency, so such clinical training should include skills that support telehealth use. Integrating structured, evidence-based digital health literacy curricula into clinical training may ensure that future health care providers develop their competency as technologies evolve.

## Supplementary material

10.2196/94722 Checklist 1 STROBE checklist.

## Abbreviations

AOR: adjusted odds ratio
DHLS: Digital Health Care Literacy Scale
NATUTS: Nurses’ Attitudes Toward Use of a Telehealth Scale
STROBE: Strengthening the Reporting of Observational Studies in Epidemiology
